# Examining a Continuous Glucose Monitoring Plus Online Peer Support Community Intervention to Support Hispanic Adults With Type 2 Diabetes: Protocol for a Mixed Methods Feasibility Study

**DOI:** 10.2196/31595

**Published:** 2022-02-24

**Authors:** Ashley H Ng, Deborah A Greenwood, Eli Iacob, Nancy A Allen, Mila Ferrer, Bruno Rodriguez, Michelle L Litchman

**Affiliations:** 1 Department of Dietetics, Human Nutrition and Sport La Trobe University Bundoora Australia; 2 Deborah Greenwood Consulting California, CA United States; 3 College of Nursing University of Utah Salt Lake City, UT United States; 4 Beyond Type 1 California, CA United States

**Keywords:** type 2 diabetes, hispanic, social support, online community, technology, peer support, diabetes, T2D, continuous glucose monitoring, behavior change, patient education

## Abstract

**Background:**

Type 2 diabetes is twice as likely to affect Hispanic people than their White counterparts. Technology and social support may be an important part of behavior change. In this study, we address gaps in diabetes care for Hispanic Spanish-speaking people with diabetes through an online peer support community (OPSC) pilot intervention using Hispanic Spanish-speaking peer facilitators with diabetes to enhance the use of continuous glucose monitoring (CGM) for diabetes management.

**Objective:**

This study aims to address gaps in diabetes care for Hispanic Spanish-speaking people with diabetes through an OPSC pilot intervention using Hispanic Spanish-speaking peer facilitators with diabetes to enhance the use of CGM for diabetes management.

**Methods:**

A mixed-methods, pre-post test design will be used in this feasibility study. A total of 50 Hispanic participants with type 2 diabetes willing to wear a continuous glucose monitor for 13 weeks will be recruited. Hispanic Spanish-speaking peer facilitators with diabetes and experience wearing a continuous glucose monitor will be employed and undergo training. Peer facilitators will help participants learn how CGM data can inform behavior changes via an OPSC. Participants will interact with the private OPSC at least three times a week. Weekly questions and prompts derived from the Association of Diabetes Care and Education Specialists, previously American Association of Diabetes Educators, and seven self-care behaviors will be delivered by peer facilitators to engage participants. Measures of feasibility and acceptability will be determined by the percentage of participants who enroll, complete the study, and use CGM (number of scans) and objective metrics from the OPSC. Efficacy potential outcomes include change in time in range of 70 to 180 mg/dL from baseline to 12 weeks, A_1c_, diabetes online community engagement, self-efficacy, and quality of life. Additionally, semistructured exit interviews will be conducted.

**Results:**

Funding for this project was secured in November 2018 and approved by the institutional review board in April 2019. Peer facilitator recruitment and training were undertaken in the second half of 2019, with participant recruitment and data collection conducted in January and April 2020. The study has now concluded.

**Conclusions:**

This study will generate new evidence about the use of an OPSC for Hispanic Spanish-speaking patients with diabetes to make behavior changes incorporating feedback from CGM.

**Trial Registration:**

ClinicalTrials.gov NCT03799796; https://clinicaltrials.gov/ct2/show/NCT03799796

**International Registered Report Identifier (IRRID):**

RR1-10.2196/31595

## Introduction

### Background

Hispanic Spanish-speaking individuals are at high risk for type 2 diabetes (T2D) and associated morbidity and mortality [1]. Hispanic populations may not receive or be able to access culturally appropriate diabetes care, which contributes to poor health outcomes [2]. Several studies have demonstrated that the use of culturally appropriate diabetes education programs improves components of T2D management, including healthy eating and increased physical activity in Hispanic Spanish-speaking individuals [3,4]. With diabetes education programs moving toward the online space, there is a dearth of research to support an online culturally appropriate initiative for Hispanic Spanish-speaking individuals to support their diabetes management [5].

### Diabetes Technology

Advances in technology are rapidly changing the diabetes landscape. One such tool is continuous glucose monitoring (CGM). Abbott Freestyle Libre is the only CGM that is available in Spanish in the United States [6]. CGM includes an interstitial glucose sensor worn on the upper arm for 14 days and a reader or smartphone app that stores glucose values for interpretation [6]. Individuals scan the reader or smartphone over their sensor to receive their glucose level history, current glucose level, and projected glucose trend using a series of arrows [6]. At present, most insurance plans in the United States cover the cost of CGM when individuals are using insulin or have substantial hypoglycemic unawareness. However, research suggests that the use of CGM results improves clinical outcomes in people with T2D and can replace finger prick self–blood glucose monitoring [7]. Furthermore, an increased number of CGM scans are associated with improved clinical outcomes such as decreased A_1c_ levels and improvements in glucose time in range [8]. Access to sensor data has been found to facilitate behavior change, possibly by creating opportunities to see the real-time impact of food, activity, and other day-to-day activities on glucose levels [7].

### Online Peer Support and Diabetes Technology

Ongoing support from peers with diabetes or health care providers is an important diabetes management strategy [9,10]. An umbrella review of face-to-face and technology-mediated peer support indicates peer support can improve A_1c_, blood pressure, and weight [11]. In addition, there is emerging evidence that online peer support community (OPSC) use can positively influence A_1c_ as well [12-14]. Similarly, participation in a diabetes OPSC is associated with clinical, behavioral, and psychosocial benefits [15]. However, little is known about the uses, benefits, and limitations of online peer support, particularly within the Hispanic Spanish-speaking diabetes population, in the context of learning how to use diabetes technology such as CGM. Our previous work indicates that Hispanic individuals desire peer interactions to relate and understand the variables that impact T2D [16]. Although 80% of Hispanic Spanish-speaking adults use the internet via a smartphone [17], it is unknown how prevalent OPSC use is in this population.

Our Patient-Centered Outcomes Research Institute (PCORI)–funded preliminary work indicates that Hispanic people are willing to use diabetes technology such as CGM if it supports the Spanish language [16]. We propose to address gaps in diabetes care for Hispanic individuals by conducting a combined CGM + OPSC pilot intervention that will use Hispanic Spanish-speaking peer facilitators with diabetes to augment health behaviors. The preliminary research design was developed using a co-design and community-based participatory research process during a separate 3-year PCORI pipeline-to-proposal award and the creation of the Intercultural Diabetes Online Community Research Council to address patient-centered concerns and priorities [18]. Investigating culturally appropriate education and support to increase use of CGM in Hispanic Spanish-speaking individuals will lead to improved clinical and behavioral outcomes.

### Study Objectives

This study aims to:

- Evaluate the acceptability and feasibility of a CGM + OPSC intervention for Hispanic Spanish-speaking individuals with T2D
- Explore the relationship between engagement in a CGM + OPSC intervention with clinical and behavioral outcomes

## Methods

### Sample Population

A mixed methods, pre-post test design will be used to evaluate the study aims. Participants will be recruited from primary care and endocrinology clinics in Utah with the aid of a research assistant. Inclusion and exclusion criteria are detailed in Textbox 1.

**Participant inclusion and exclusion criteria.**

**Inclusion criteria**

- Adults 21 years and older
- Clinical diagnosis of type 2 diabetes
- Self-report as Hispanic
- Ability to communicate fluently in Spanish
- A_1c_ ≥8% as per clinic records
- Willing to wear an intermittently scanned continuous glucose monitor for 14 weeks
- Access to the internet and willing to engage in an online peer support community
- Willing to avoid vitamin C 500 mg and aspirin 325 mg or greater daily due to interference with intermittently scanned continuous glucose monitoring accuracy

**Exclusion criteria**

- Currently using insulin for diabetes management
- Use of continuous glucose monitor or intermittently scanned continuous glucose monitor in previous 6 months
- Current participation in other diabetes clinical trials
- Alcohol or drug abuse or dependent
- Severe illness (physical or mental health)
- Cognitive impairment
- Current use of high dose steroids
- Hospitalization more than twice in the past 12 months or other impairment that would, in the opinion of the investigators, interfere with their ability to complete the study
- Pregnant or planning to become pregnant during the study
- Uncorrected hearing or vision impairment
- Life expectancy less than 6 months

### Sample Size Calculation

Power and sensitivity analyses were conducted in GPower [19] using differences between means for time in range, A_1c_, and online peer support engagement coded as a continuous variable. Assuming an alpha of .05, a sample size of 43 is sufficient to provide 90% power to detect a medium effect size (Cohen *d* 0.5). A total of 50 participants will be recruited to allow for a 14% dropout rate (86% completion rate), which is in line with a 20% attrition rate from a previous study involving Hispanic participants living with diabetes [20] and a 10% attrition rate from a study investigating young adults with diabetes trialing an online peer support intervention [21]. All participants will be invited to complete the qualitative interview with an aim of a 50% (n=25) participation rate, which is expected to be adequate to reach data saturation.

### Recruitment

Recruitment will take place between January and April 2020. Experienced community health workers with previous success in recruiting Hispanic Spanish-speaking participants will be used. Opt-out letters will be sent to patients seen by bilingual physicians and physician assistants at an academic community clinic. Those who do not opt out will be contacted by the research assistant. Providers and federally qualified community health clinics serving the Hispanic Spanish-speaking population and diabetes specialty offices will be contacted to refer patients to participate in the study. Participants will be contacted by phone and screened for eligibility as outlined in Textbox 1. Those who meet study inclusion criteria will be invited to a convenient community location accessible to the participant, such as a private room in a library, or a video teleconferencing platform to meet with the research assistant. The research assistant will describe the study, go over the consent form with the participant, and answer questions participants may have. If the individual consents to participate, they will sign the consent form, complete the baseline surveys, and be provided a 7-day blinded version of CGM, Libre Pro [22], to establish a baseline glucose profile prior to the intervention (study visit 1). The research assistant will collect an A_1c_ (via home testing kit) and gather clinical information such as height, weight, and blood pressure. At study visit 2, the blinded CGM will be collected, and the participants will start both CGM and the OPSC. Participants are expected to be active within the OPSC for 12 weeks upon study enrollment. Participants will be enrolled on a rolling basis over a 3-month period. The online community will be active for a 6-month period. After 12 weeks, participants are welcome to continue using the OPSC.

### Peer Facilitator Recruitment and Training

We will recruit and employ 5 Hispanic Spanish/English bilingual peer facilitators who live with diabetes with experience using CGM. Individuals identified by Hispanic Spanish-speaking leaders from our PCORI community advisory board will be emailed information about the position and eligibility criteria (Multimedia Appendix 1). Interested individuals will complete a survey to confirm they meet the eligibility criteria. Successful applicants will be interviewed via teleconference with at least two members of the research team to confirm suitability for the role and to answer any questions they may have. Successful peer facilitators will sign a contract that outlines the expectations of their role prior to training completion.

Peer facilitators will be required to undertake three online training sessions: the Association of Diabetes Care and Education Specialists (ADCES), previously known as American Association of Diabetes Educators, level 1 paraprofessional training; a study-specific training; and training to use the OPSC platform. The goal of the training sessions is to provide peer facilitators with strategies to build relationships with participants as a support person who understands their challenges. The empowerment approach [23] and motivational interviewing [24] techniques will be used to build self-confidence and self-efficacy in peer facilitators to support goal setting, problem-solving, and behavior change in participants. Ultimately, the goal is to help participants increase their time spent within the target glucose range of 70 to 180 mg/dL. Individuals will be most successful when they set their own self-management goals and establish a plan that fits within their schedule and life [24]. Peer facilitators will use empowering person-first language following the American Diabetes Association and ADCES guidelines [8,25].

The ADCES Level 1 paraprofessional training consists of self-directed coursework including reading practice documents and participating in webinars totaling 14.5 hours [26]. Coursework content includes basic diabetes information for nonclinicians, teaching and learning in diabetes education, cultural competency, goal setting, motivational interviewing, use of person-centered language, delaying diabetes-related complication development and progression, and learning from people living with diabetes.

The study-specific training familiarizes peer facilitators to the research study; the preferred tools used to build and engage the OPSC; and strategies to maintain their own health, mental well-being, and online safety. A key focus of the trainings is to reduce stigma associated with T2D and to foster an understanding that, although there are genetic factors that cannot be changed, there are actions people can take to feel healthy, have a high quality of life, and reduce their risk of developing diabetes-related complications. Peer facilitators were trained to not provide medical advice and to refer participants back to their primary care provider if they have specific questions about medications or experienced out of range glucose levels.

The OPSC will also be reviewed by a Spanish/English bilingual diabetes care and education specialist on a regular basis. Feedback on coaching techniques or diabetes information will be provided during three virtual meetings with the peer facilitators.

### Online Peer Support Community Intervention

The OPSC will be hosted on EsTuDiabetes (EsTuDiabetes.org), a program of Beyond Type 1 (BeyondType1.org), which includes a host of discussion forums. Participants and peer facilitators will have a profile with a username of their choosing. The private discussion forum will only be accessible by the research team and participants. Peer facilitators will be scheduled to monitor the OPSC hourly over two main shifts of 6 AM to 3 PM and 3 PM to 12 PM.

Participants will be encouraged to interact with the online group at least three times a week at a time that is most convenient to them. A priori weekly questions and prompts to facilitate discussion and topics covering the ADCES 7 self-care behaviors [27], frequency of CGM scanning, and how to analyze glucose patterns will be scheduled. The weekly schedule will follow the format presented in Textbox 2. These topics will repeat every 12 weeks given the rolling enrollment.

Peer facilitators will engage in weekly personal experiments along with participants and will share their personal goals, experiences, challenges, and successes during the study. Table 1 describes the cycle of weekly discussion topics and examples of personal experiments and its translation into Spanish. As content will be driven by participants’ individual experiences and is not time oriented, this allows new members to join the online peer support group at any day of the week during the intervention period.

**Weekly prompt and activity schedule outline.**

**Monday**

Development of a “personal experiment” focused on behavior change relevant to the weekly theme

**Wednesday**

Group poll question focused on personal experiment progress

**Friday**

Group “check-in” using a Likert scale to assess goal achievement for the week

**Table 1:** Examples of possible weekly personal experiments to be discussed (posted in Spanish).

Week	Topic	Examples of personal experiment prompts
1	Healthy eating	I will scan my Libre before and 2 hours after breakfast every day for one week to see how my glucose changes. *Chequearé mi libre antes y 2 horas después del desayuno todos los días durante una semana para ver cómo cambia mi glucosa.*
2	Being active	I will scan my libre before and 2 hours after walking or exercise every day for one week to see how my glucose changes. *Escanearé mi libre antes y 2 horas después de caminar o hacer ejercicio todos los días durante una semana para ver cómo cambia mi glucosa.*
3	Healthy sleep	I will scan my libre before bed and after waking up in the morning for one week to see how my glucose changes with hours of sleep. *Escanearé mi libre antes de acostarme y después de despertarme por la mañana durante una semana para ver cómo cambia mi glucosa según las horas que duermo.*
4	Healthy coping	I will scan my libre before and 2 hours *after a stressful event* for one week to see how my blood glucose changes. *Escanearé mi libre antes y 2 horas después de un evento estresante durante una semana para ver cómo cambia mi glucosa.*
5	Healthy eating	I will post a question every day to the online group asking for suggestions for healthy snacks I can take to work for one week. *Publicaré una pregunta todos los días al grupo pidiendo sugerencias sobre tentempiés o botanas saludables que pueda llevar al trabajo durante una semana.*
6	Being active	I will post a question every day to the online group sharing the physical activity I am doing during the workday to increase steps. *Publicaré una pregunta todos los días al grupo compartiendo la actividad física que estoy haciendo durante un día de trabajo para aumentar los pasos.*
7	Healthy sleep	I will stop looking at screens one hour before bed every night to increase my hours of sleep. *Dejaré de mirar las pantallas una hora antes de acostarme todas las noches para aumentar mis horas de sueño.*
8	Healthy coping	I will use a meditation app once a day for 10 minutes to focus on stress reduction. *Usaré una aplicación de meditación una vez al día durante 10 minutos para enfocarme en disminuir el estrés.*
9	Healthy eating	I will try a new vegetable this week. *Probaré una nueva verdura esta semana.*
10	Being active	I will ride my bike to work 3 days a week. *Montaré en bicicleta al trabajo 3 días a la semana.*
11	Healthy sleep	I will stop drinking coffee by 2pm every day this week. *Dejaré de tomar café a las 2pm todos los días esta semana.*
12	Healthy coping	I will take a bath at night before going to bed and relax for 20 minutes each night. *Me bañaré por la noche antes de acostarme y me relajaré durante 20 minutos cada noche.*

### Participant Timeline

**Figure 1:**
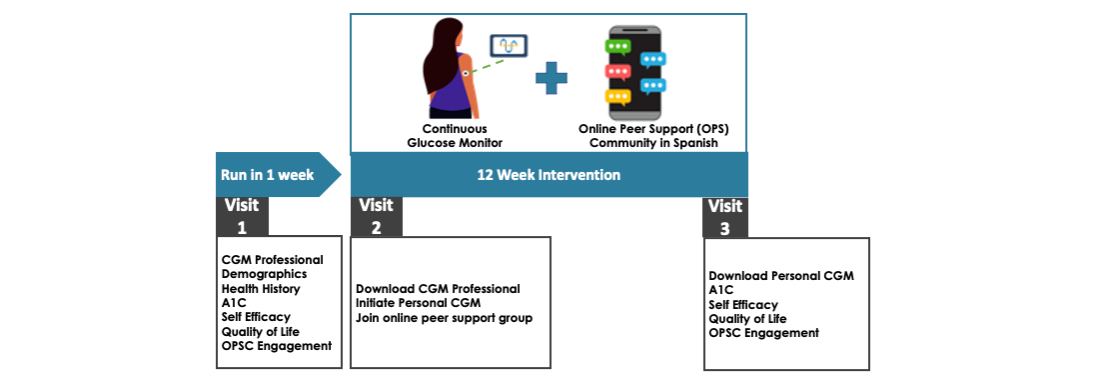
Study timeline. CGM: continuous glucose monitoring; OPSC: online peer support community.

Each participant will be enrolled in this study for 13 weeks, inclusive of a run-in period of 1 week, and will follow the timeline as per Figure 1.

### Data Management

Screening tracking, enrollment, and survey data will be collected and stored via REDCap [28], a secure Health Insurance Portability and Accountability Act–compliant data collection software hosted at the University of Utah. Surveys will include those assessing demographics, clinical history, and study measures. All individuals involved in screening and data entry will be trained to use the database.

### Outcome Measures and Planned Data Analyses

The main components of planned descriptive and statistical analyses are separated into two study aims. Study aim 1 will focus on feasibility and acceptability using objective metrics and semistructured interviews. Analyses for study aim 2 will examine the efficacy potential of the following outcomes: time in range, A_1c_, and validated surveys. An overall summary of the study outcomes and measures across data collection time points and study aims is listed in Table 2.

A description of the analytical methods are given in the following section. Existing objective metrics derived from the online peer support group platform to measure feasibility and acceptability are detailed in Table 3.

**Table 2:** Summary of study outcomes at each study time point.

Variable			Measure		Data collection			
					Study visit 1 (enrollment)	Study visit 2 at 1 week (T0)		Study visit 3 at 12 weeks (T1)
Demographics			Age, gender, education level, income level, insurance type, ethnicity, employment, married, comorbidities		✓			
Clinical: weight, height, blood pressure			Weight: lb Height: ft, in Blood pressure: systolic/diastolic		✓			
Health history			Diabetes diagnosis, year of diagnosis, previous diabetes education		✓			
Diabetes management			Self-reported		✓			
**Study aim 1: feasibility and acceptability**								
	Acceptability	Objective online engagement metrics including number of reactions or comments per post, number of views per post, and Likert scale responses to polls					✓	
	Feasibility	Attrition rates within online peer support group					✓	
	Satisfaction and participant experiences	Semistructured interviews^a^					✓	
**Study aim 2: clinical and behavioral outcomes**								
	Clinical measurements	Time in range^b^		✓ (PRO CGM^c,d^ placement)		✓ (PRO CGM download, personal CGM placement)	✓ (personal CGM download)	
	Glycemic levels	A_1c_, standard CGM/isCGM data reporting [10]		✓			✓	
	Self-efficacy	Self-efficacy for diabetes [27]		✓			✓	
	Online peer support community engagement	Diabetes Online Community Engagement Survey [28]		✓			✓	
	Quality of life	WHO-5^e^ [29]		✓			✓	

^a^Semistructred interviews available in Multimedia Appendix 2.

^b^Average glucose level and number of minutes in 70 mg/dl to 180mg/dl in last 7 days of the study compared to average glucose level and number of minutes in 70mg/dl to 180mg/dl measured by initial blinded 7 days of baseline glucose data.

^c^PRO CGM: professional continuous glucose monitoring.

^d^Data is blinded to participants.

^e^WHO-5: World Health Organization Five.

**Table 3:** Online peer support group platform metrics.

Metric	Definition
Days visited *Días Visitados*	This metric reflects how many days the member has logged into the forum.
Read time *Tiempo de Lectura*	This metric reflects how much time the member has spent reading in the community since registering.
Recent read time *Tiempo de Lectura Reciente*	This metric reflects how much time the member has spent reading in the community within the last 60 days.
Topics viewed *Temas Vistos*	This metric reflects how many posts the member has clicked on but not necessarily read them.
Posts read *Publicaciones Leídas*	This metric reflects how many posts the member has actually read, based on the time they spent scrolling.
Likes given *Likes dados*	This metric reflects the number of times the member agrees, supports, and highlights posts.
Topics created *Temas Creados*	This metric reflects how many new topics the member has created within the forum.
Posts created *Publicaciones Creadas*	This metric reflects how many replies the member has given in different topics.
Likes received *Likes recibidos*	This metric expresses agreement, supports, and highlights posts with the prominent button on every post.
Lasts post *Última publicación*	This is the date of the last topic or post creation.
Last seen *Última vez visto*	This is the last time the member logged into the forum.
Views *Vistas*	This metric shows how many members have clicked on a topic.
Trust level *Nivel de Confianza*	Members will have different trust levels depending on the frequency of their participations, it goes from Trust Level 0 (new user) to Trust level 3 (regular).

### Study Aim 1 Analysis

To address feasibility and acceptability, demographics and clinical information from all participants, including noneligible, eligible but refused, eligible and enrolled, and variables related to participant dropout, will be assessed. These data will be used to create a CONSORT (Consolidated Standards of Reporting Trials) diagram detailing the feasibility of recruiting participants into the study. Descriptive statistics examining individuals that enrolled versus those that did not will provide meaningful information for future studies. Recruitment, participation, and dropouts will be reported with frequency and percentages. We will also compute 95% CIs. These intervals serve as population estimates for future larger studies. Finally, objective and anonymous summary statistics of participation within the online peer support group as highlighted in Table 3 and participant interviews will be reported.

### Study Aim 2 Analysis

The goal of study aim 2 is to examine the efficacy potential of the intervention on time in range, A_1c_, and validated surveys. REDCap facilitates an easy data export to multiple statistical software packages [28]. Data cleanup and all analyses will be done in SPSS version 25 (IBM Corp). Time in range (70-180 mg/dL) from baseline to study completion will be measured as per ambulatory glucose profile downloads. Other outcomes include A_1c_, OPSC engagement [13], self-efficacy for diabetes [29], and quality of life (World Health Organization 5 [WHO-5]) [30], which are validated scales for diverse populations available freely for research use. Linear mixed effects models will be used, as it allows for the inclusion of random intercepts (participants begin at different levels of the outcomes) and potential covariates, including age, gender, and ethnicity. Coefficient estimates and robust SEs will be reported while controlling for the covariates of interest. In keeping with an intent-to-treat standard, we will use maximum likelihood estimation, as it accommodates missing data, and thus all individuals will be included in the analysis regardless of dropout or missing data. Nevertheless, we will explore the data for potentially nonignorable missing data patterns and report on these findings. To minimize loss of scale scores due to missing items in a computed scale (eg, WHO-5 and self-efficacy scale), scores based on available items will be prorated. Data will be coded as missing if 30% of the items in a computed score have not been recorded.

### Data Monitoring and Auditing

Although the research assistant will act as the primary contact with peer facilitators, regular check-in meetings (minimum of weekly via group text messaging and every 6 weeks via video teleconferencing) will be scheduled between the research assistant, peer facilitators, and research team via videoconference. These team meetings will serve as another platform to problem solve any issues that arise and to share learnings. Repeatable instrument communication logs will be available to keep notes on interactions between the research team and participants, including the recording of any adverse events. Where feasible and appropriate, the research assistant will document reasons for participant drop out. Data safety monitoring logs will be available to monitor data discrepancies and opportunities for double-checking data entry.

Fidelity to the intervention will be assessed independently through an initial weekly review using a predetermined checklist (Multimedia Appendix 3) of the online content by bilingual clinical advisors to ensure that concepts of empowerment and motivational interviewing are incorporated, followed by ongoing periodic spot reviews.

### Ethics and Dissemination

Approval from the University of Utah Institutional Review Board (IRB) was obtained in April 2019 (IRB 00125233). Protocol modification will undergo additional IRB approval, be communicated to the research team, and be updated on the clinical trials registry, including:

- How personal information about potential and enrolled participants will be collected, shared, and maintained to protect confidentiality before, during, and after the trial
- Financial and other competing interests for principal investigators for the overall trial and each study site
- Statement of who will have access to the final trial data set, and disclosure of contractual agreements that limit such access for investigators
- Provisions, if any, for ancillary and posttrial care, and for compensation to those who experience harm from trial participation

If participants self-report that their glucose levels have been over 270mg/dL for 2 weeks, they will be encouraged to visit their primary care provider. Peer facilitators will reach out to the research team with any concerns about participants. To ensure the safety of peer facilitators, they will not have contact with participants beyond the online peer support group. Behavior expectations and guidelines will be posted and pinned to the top of the online peer support group page to guide appropriate interactions and consequences if these are breached. Throughout the study, peer facilitators are expected to continue with their own health and diabetes management, and notify the research team if health issues arise that may prevent them from fulfilling their role.

## Results

Funding for this project was secured in November 2018. At the time of reporting, peer facilitator employment and training has been completed with participant recruitment commenced in January 2020 through to April 2020.

The main findings from the study will be presented in conferences and reported in peer-reviewed publications. The study has concluded.

## Discussion

The aim of this study is to pilot and test the efficacy potential of the use of an OPSC with the use of peer facilitators to provide culturally appropriate education and support for Hispanic Spanish-speaking people with T2D in their diabetes self-management. The use of a culturally appropriate online peer support group and peer facilitators within the Hispanic Spanish-speaking population with T2D is expected to provide convenient access to informational, appraisal, and emotional support for diabetes self-management. It is anticipated that additional support provided by peer facilitators in the online peer support group will encourage positive health behavior change in this population who are at higher mortality risk from T2D compared to their White counterparts.

Foreseeable challenges within this pilot trial include recruitment and retention of eligible participants and peer facilitators. In anticipation, we have used community health workers and bilingual staff to support recruitment and retention efforts. The PCORI Advisory Board will help identify and recruit peer facilitators.

The SPIRIT (Standard Protocol Items: Recommendations for Interventional Trials) Checklist [31] was used to ensure quality reporting and consideration of all standard protocol items recommended for international trials.

## Appendix

Multimedia Appendix 1 Peer facilitator position description and eligibility criteria.

Multimedia Appendix 2 Interview schedule.

Multimedia Appendix 3 Peer facilitator fidelity review.

## Abbreviations

ADCES: Association of Diabetes Care and Education Specialists
CGM: continuous glucose monitoring
COSORT: Consolidated Standards of Reporting Trials
IRB: Institutional Review Board
OPSC: online peer support community
PCORI: Patient-Centered Outcomes Research Institute
SPIRIT: Standard Protocol Items: Recommendations for Interventional Trials
T2D: type 2 diabetes
WHO-5: World Health Organization 5
