# Supplementary material for: Examining a Continuous Glucose Monitoring Plus Online Peer Support Community Intervention to Support Hispanic Adults With Type 2 Diabetes: Protocol for a Mixed Methods Feasibility Study
Source: JMIR Res Protoc. 2022 Feb 24;11(2):e31595. doi: 10.2196/31595 (PMC8914754; doi:10.2196/31595)
Supplement: Multimedia Appendix 1 [file resprot_v11i2e31595_app1.docx]

**Multimedia Appendix 1. Peer Facilitator Job Description**

PURPOSE:

The peer facilitator is an important research team member, engaging directly with all research study participants via the online peer support group and assisting other investigators and team members. The position performs a variety of roles that include:

- Welcoming participants to the peer support group
- Orienting them to the 12-week study discussions
- Offering a variety of discussion points relating to living well with diabetes
- Assisting participants in articulating their goals for health and well-being
- Helping participants to learn and practice new self-care skills
- Helping participants monitor their progress
- Assisting participants in learning how to interpret isCGM data
- Helping participants make positive health behaviour choices related to the AADE 7 self-care behaviours
- Supporting participants to advocate for themselves when speaking with their healthcare team

RESPONSIBILITIES:

The position performs a wide range of tasks to assist participants. The position requires that the peer facilitator establish an online, ongoing relationship with study participants.

The peer facilitator provides information and advocates for participants’ personal health goals and plans, by partnering with participants via a closed, private online peer support group designed for the study. The peer facilitator will help participants navigate the online peer support group, encourage them to ask questions of others and, as they become more comfortable, provide support through answering questions from other participants.

The peer facilitator assists the participants to explore personal health choices and understand how those choices impact their diabetes management. The peer facilitator will encourage participants to identify weekly personal goals and to engage in “personal experiments” to identify potential actions in self-care areas, and identification of support and follow-up to implement a plan.

Utilizing strong relational and facilitation skills, the peer facilitator acts as a point of contact to navigate through a 12-week peer support process for participants.

CONTRIBUTION:

The peer facilitator must adhere to the highest standards of compassion, commitment, excellence, professionalism, integrity, accountability, and stewardship.

ELIGIBILITY & QUALIFICATIONS:

This position is ideal for those who have an interest in working directly with others living with diabetes to share your successes and challenges. Excellent interpersonal skills with a positive and upbeat online presence are a prerequisite as this position involves constant online contact with participants. Individuals interested in this position must be bilingual in English and Spanish, live with diabetes, wear a personal CGM device/isCGM, and complete a two-part peer facilitator training online. At no time will a peer facilitator provide medical advice, medication advice or answer questions that are beyond the scope of the role. Peer facilitators will use techniques including motivational interviewing or peer coaching, rather than directing or telling participants what they need to do. More information and strategies that incorporate motivational interviewing will be covered in the peer facilitator training. The role of a peer facilitator is that of a coach, cheerleader, support person, empathetic listener, and someone who can share their lived experience.

SUPERVISORS:

Peer facilitators will be supervised by the Research Assistant (who is also bilingual) who will report back to the research team. A member from the research team will be your key peer facilitator mentor with other research team members providing training and oversight of the study.

TIME COMMITMENT:

The initial peer facilitator training will be provided at no cost to you and you will be provided with an American Association of Diabetes Educators Level 1 Paraprofessional Certificate at the completion of training. The online AADE training will take approximately 14.5 hours to complete and must be completed by a date specified by the research team prior to engaging with participants. An additional study specific online training will take approximately 2 additional hours to complete.

The peer facilitator will be required to attend monthly research team meetings and ad hoc meetings as necessary to discuss the study procedures and the online peer support group progress. A schedule will be developed so peer facilitators know when they need to be available online. Each peer facilitator will be assigned 14 shifts per month over six months to be “on call” during assigned hours. During a typical day, there are two shifts (6am - 3pm and 3pm to 10pm Mountain Time) it is expected that the peer facilitator will check the online peer support group approximately every 2 hours to post messages, respond to comments and questions and to encourage and support participants. The amount of time spent within the online peer support group will vary based on study group size, time of day, day of week etc. The peer facilitator will keep a log of the time they engage and will “sign off” each shift with a mini handover report for the peer facilitator who is taking over the next shift. A template will be provided for peer facilitators to assist with the handover process.

Every Monday the research assistant will post a “Monday Motivator” challenge designed ahead of time by the research team that includes 4 topics in rotation over each week during a month: healthy eating, being active, healthy sleep and healthy coping/stress management. Peer facilitators will encourage participants to engage in a weekly “personal experiment” to make SMART goals towards positive behaviour change. In addition, weekly poll questions and check-ins will be distributed on Wednesday and Friday respectively for participant completion. The research team will be available to peer facilitators should they have any questions, require guidance or suggestions to encourage participation.

BENEFITS:

Peer facilitators will receive a stipend for 7 months (this includes one month of training and 6 months of intervention) after submitting an invoice documenting their time engaged in the online peer support group. After completion of the peer facilitator training, they will be eligible to receive the American Association of Diabetes Educators Level 1 Paraprofessional Certificate.
