# Supplementary material for: Examining a Continuous Glucose Monitoring Plus Online Peer Support Community Intervention to Support Hispanic Adults With Type 2 Diabetes: Protocol for a Mixed Methods Feasibility Study
Source: JMIR Res Protoc. 2022 Feb 24;11(2):e31595. doi: 10.2196/31595 (PMC8914754; doi:10.2196/31595)
Supplement: Multimedia Appendix 2 [file resprot_v11i2e31595_app2.docx]

**Multimedia Appendix 2. Interview Schedule**

1. Can you tell me about your experiences in using the Freestyle Libre and being part of the online peer support group?

- Prompt: Did use of Libre help you identify relationships between use of tech and making behaviour changes
- If you were able to access the Libre, would you see yourself continuing to use the it? If so, how often?

1. Tell me about your experiences of working with the peer facilitator
   - Prompt: learning about how food choices, physical activity, stress and sleep impact glucose trends
   - Technology troubleshoot-Did the peer support help you keep using the Libre?
2. Please tell me what changes you have made to your diabetes management from this study?

- Prompt: food choices, physical activity, stress and sleep
- What changes will you likely keep/stick to after this study?
- How did the personal experiments help you make changes?

1. Tell me how this study has changed the way you use social media

- Have you supported others via the online peer support group?

1. What have you enjoyed from this study?
   - What worked for you, what didn’t
2. What recommendations would you suggest to improve this support service?
3. Would you recommend this online peer support group or other resources on EsTuDiabetes.org to other people with type 2 diabetes who are learning to use the Freestyle Libre? (Provide a score from 1-10 - link to net promoter score)
   - Why?
   - Would you like to continue to engage in the online peer support group?
4. Is there anything else you would like to say about using the online peer support group (with Peer Facilitators) in combination with a Libre to support your diabetes management that we have not covered?
