# Supplementary material for: Examining a Continuous Glucose Monitoring Plus Online Peer Support Community Intervention to Support Hispanic Adults With Type 2 Diabetes: Protocol for a Mixed Methods Feasibility Study
Source: JMIR Res Protoc. 2022 Feb 24;11(2):e31595. doi: 10.2196/31595 (PMC8914754; doi:10.2196/31595)
Supplement: Multimedia Appendix 3 [file resprot_v11i2e31595_app3.docx]

**Multimedia Appendix 3. Peer Facilitator (PF) Fidelity Review**

Please use the checklist below to review the forum posts.

**Week: Date:**

| **Competency** | **Y/N** | **Comments** |
| --- | --- | --- |
| Were the 3 main prompts posted (Mon, Wed, Fri)? |  |  |
| Were new participants welcomed to the group? |  |  |
| Did PFs encourage conversation with and among the group? |  |  |
| Were there any questions from participants that were unanswered? |  |  |
| Was the discussion from the PFs in a positive, encouraging, motivational interviewing method using person first language? |  |  |
| Were there any medical issues discussed that should be referred to an MD? |  |  |
| Did PFs work in similar ways?  Are there suggestions for them? Is one approach working better than others to encourage participation? |  |  |
| Do we need a PF group call for you to provide input or do we send email/letter/report? |  |  |
| How and when do you think the next review is needed? |  |  |
| Other items identified or things we should do differently? |  |  |
| Any specific feedback for individual PF? |  |  |
